# A Unique Presentation of Acute Kidney Injury With COVID-19

**DOI:** 10.7759/cureus.19381

**Published:** 2021-11-08

**Authors:** Priyata Dutta, Sulagna Das, Adam Fershko

**Affiliations:** 1 Physiology, University of Michigan, Ann Arbor, USA; 2 Internal Medicine, Kettering Medical Center, Kettering, USA; 3 Internal Medicine, Kettering Medical Center, Dayton, USA

**Keywords:** creatinine, intensive care unit, hemodialysis, acute kidney injury, covid 19

## Abstract

Although the respiratory system is the primary target of COVID-19 pneumonia, it can also notably affect the other systemic organs such as renal and cardiac. The incidence and prevalence of SARS CoV-2 associated acute renal failure are emerging day by day. While the pathogenesis is not clearly understood, it is considered multifactorial. Initially, the COVID-19-associated renal dysfunction was limited to acute tubular injury. However, over time a wide spectrum of clinical manifestations has been reported. Therefore, prompt investigation and early initiation of supportive treatment can potentially reduce the mortality and morbidity associated with this systemic disease. In this case report, we present a unique presentation of a COVID-19 with acute kidney injury where the patient was admitted to the intensive care unit with clinical features of acute renal failure with concomitant diagnosis of COVID-19, unlike other reported cases where patients were admitted to the intensive unit with respiratory distress and subsequently developed renal failure.

## Introduction

The rapid emergence of the novel coronavirus has become a global public health crisis. The novel coronavirus is now known as the severe acute respiratory syndrome coronavirus 2 (SARS-CoV-2). In March 2020, the World Health Organization (WHO) declared the COVID-19 outbreak a pandemic [1]. A wide spectrum of clinical manifestations of COVID-19 ranges from asymptomatic or mild infection such as flu-like fever, myalgia, cough, sneezing, mild pneumonia to rapidly progressive acute respiratory distress syndrome (ARDS), as well as potential multi-organ complications including encephalopathy, cardiac arrhythmia, thromboembolism, and acute renal failure [2]. Most of the intensive care admissions develop COVID-19-associated ARDS often requiring mechanical ventilation and the mortality rates are often higher among these patients owing to multi-organ failure. In the case of the kidney, there is a need for renal replacement owing to the incidences of AKI [3]. The incidence of AKI was about 6.7% in the last SARS epidemic [4]. However, 5%-25% of hospitalized patients with COVID-19 now develop acute kidney injury (AKI) and among them, 5%-15% end up in renal replacement requirement therapy [2,5]. Patients with an elevated baseline of creatinine (baseline creatinine 0.74 to 1.35 mg/dL) are more prone to develop AKI than patients with a normal baseline of creatinine [6]. Although the mechanism of renal effect is not clear, it has been suggested that different mechanisms are responsible for developing AKI in COVID-19 patients [2]. Clinical manifestation resulting from COVID-19-induced renal function impairment ranges from hematuria, proteinuria, oliguria, and AKI subsequently resulting in a high rate of mortality and morbidity compared to COVID-19 cases without AKI. Herein, we present a case of an African American man who was admitted to the intensive care unit (ICU) with the symptoms of AKI secondary to COVID-19 pneumonia followed by complete recovery.

## Case presentation

A 46-year-old African male with a past medical history of essential HTN presented to the emergency department with a five days history of cough, shortness of breath, diarrhea, muscle cramping, fatigue, poor oral intake and decreased urinary output. The patient was tested positive for COVID-19 one day prior to admission. On further examination, the patient was febrile, hemodynamically stable with a blood pressure of 125/57 mmHg with a mean arterial pressure of 77 mmHg, heart rate of 83 bpm, respiratory rate of 24 per minute, and oxygen saturation was 93%. The patient was adequately oxygenated on a 2 L nasal cannula. Initial lab report revealed 133 meq/L of Na, chloride 88 meq/L, potassium 6.3 meq/L, calcium 8 meq/L, creatinine 23 mg/dL, BUN 195 mg/dL, creatinine kinase 1,200 U/L, lactate dehydrogenase (LDH) 212 U/L, C-reactive protein 126.6, and elevated D-dimer 4,433 (Table 1). Arterial blood gas showed bicarbonate 6 meq/L, CO_2_ 6 mmol/L, anion gap 41 mmol/L, PH 7.17. His liver function panel was normal. Urinary analysis showed protein >600 mg/dL, blood 1+, creatinine 404.6 mg/dL, and urine protein electrophoresis was 1,735 mg.

**Table 1:** Lab values showing the serum creatinine, anion gap, and serum CO2.

	Ref ranges and units	Day one	Day two	Day three	Day four	Day five
Creatinine	0.7-1.3 mg/dL	23 mg/dL	15 mg/dL	9 mg/dL	4 mg/dL	3.5 mg/dL
Anion gap	7-16 mmol/L	41 mmol/L	24 mmol/L	13 mmol/L	14 mmol/L	12 mmol/L
CO_2_	21-31 mmol/L	6 mmol/L	12 mmol/L	35 mmol/L	30 mmol/L	20 mmol/L

**Figure 1:**
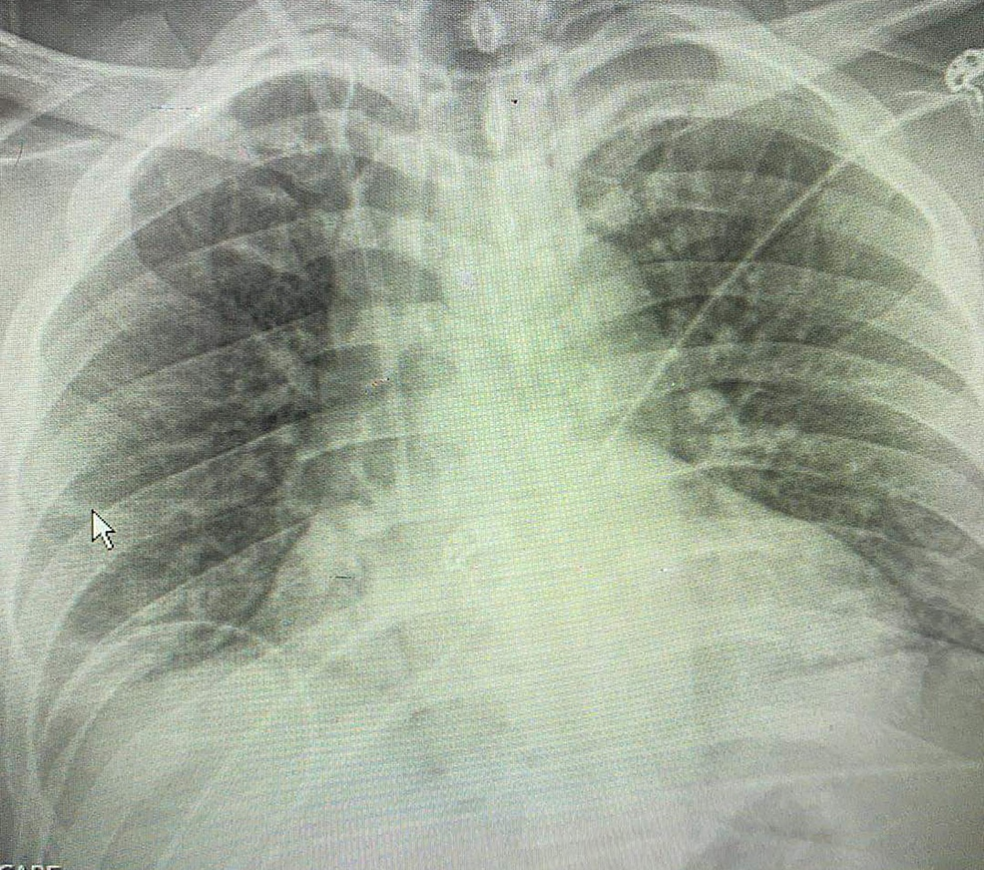
Chest x-ray showing the non-acute phase of COVID-19 pneumonia.

EKG findings were non-significant for hyperkalemia, Chest x-ray was negative for the acute process of viral infection (Figure 1). Since the patient was dehydrated he was started on 2 L of normal saline followed by 1 g of calcium gluconate. Repeat potassium was 7.7 meq/L. Therefore, the patient was admitted to the COVID ICU followed by a nephrology consultation. Since remdisivir is not a good drug of choice in renal dysfunction, 6 mg of decadron was started and sodium bicarbonate drip was started at 150 mL/hr. Additional investigation including color Doppler ultrasound of lower extremities was done due to high D-dimer and was negative for deep vein thrombosis. Ultrasound of kidney and urinary bladder was also negative for hydronephrosis. Despite the lack of improvement in supportive treatment, the patient was scheduled for hemodialysis. Subsequently his renal function and electrolytes status improved. Creatinine decreased to 3.1 mg/dL, creatinine kinase 895 IU/L, and the anion gap trended to 15. On discharge, potassium was 4.4 meq/L, creatinine was 2.9 mg/dL and he was sent home on room air with four more days of decadron and pulmonary rehabilitation exercise was done to enhance the recovery from COVID-19 pneumonia.

## Discussion

During the early stage of the COVID-19 pandemic, the incidence of COVID-19-related AKI was very minimal [7]. However, nowadays its prevalence is increasing day by day, especially among ICU patients with COVID-19. It has been reported that only 5% of the hospitalized COVID-19 patients develop renal complications and among them, 40% need intensive care [8]. The mortality was measured at 40.8% among patients on invasive mechanical ventilation. However, the mortality rate increased to 71.6% for patients on invasive mechanical ventilation, vasoactive drugs, and requiring renal replacement therapy [3]. AKI is evaluated as a bad prognostic factor and indicator of disease progression [8]. Another study reveals that the prevalence is much higher in the male black population and according to the Intensive Care National Audit and Research Center (ICNARC), the COVID-19 death rate is relatively higher in Asian and black patients who are admitted to the ICU [4]. Therefore, AKI has become a common complication of COVID-19 pneumonia. The clinical manifestation of renal dysfunction ranges from mild proteinuria to advanced renal complications from acute renal failure [8].

The pathophysiology behind this complication is yet to be clearly understood. Available evidence suggests that the pathogenesis is multifactorial. Both direct and indirect mechanisms are responsible for COVID-19-related AKI [7]. This dysfunction can manifest in various ways ranging from pre-renal injury resulting from hypovolemia or cardio-pulmonary syndrome to acute tubular injury and APOL1 (Apolipoprotein L1) genetic susceptibility [7]. The pathophysiology behind the direct renal tubular injury results from disruption of the angiotensin-converting enzyme-2 (ACE-2) pathway, which subsequently results in acute tubular injury, proteinuria, collapsing glomerulopathy, and dysfunction of mitochondria. This theory has been supported by the presence of viral particles in proximal convoluted renal tubules and podocytes reported in a study on 26 patients dying of COVID-19 [7,8]. In addition, genetic polymorphism of the APOL1 gene is also considered a high-risk genetic variant for developing collapsing glomerulopathy- a morphological variant of focal segmental glomerulosclerosis in African American descent or black population [7]. In addition to direct mechanism, an indirect pathway has also been discussed in a few studies which include the immunological association of COVID-19 and cytokine storm induced by a viral infection that results in hypoxia, shock, sepsis, rhabdomyolysis, and subsequently multiorgan failure including renal failure and death. Finally, microthrombi can also play a potential role in COVID-19-induced renal dysfunction [7].

Acute renal impairment such as pre-renal injury may arise from the COVID-19-associated systemic effects such as insensible fluid loss through vomiting and diarrhea which is a common clinical manifestation of COVID-19 pneumonia. Sometimes acute tubular injury or acute intestinal nephritis can occur as a consequence of using nephrotoxic drugs or more specifically antibiotics as a part of the patient management care [7]. Furthermore, those who develop a secondary infection due to viral or bacterial etiology are more prone to develop sepsis-related AKI [7]. Association of multiple comorbidities including chronic kidney disease, essential hypertension, diabetes mellitus, coronary heart disease, and advanced age is also strongly correlated with the development of AKI in COVID-19 patients [7]. Since inflammation plays a potential role in the COVID-19-associated pathogenesis, anti-inflammatory drugs are found to be useful in the mitigation of complications related to COVID-19. A prospective meta-analysis of clinical trials showed corticosteroid is beneficial in the reduction of mortality rate and complications associated with COVID-19 compared to the patients, not on corticosteroid [9]. It has also been reported in another trial that patients on dexamethasone did not require renal replacement therapy compared to patients with placebo [10]. Furthermore, it also demonstrated that the use of biological agents like tocilizumab, an IL-6 receptor antibody is also associated with reduced incidence of AKI-associated COVID-19 pneumonia [10].

Regarding the management, supportive therapy is the mainstay of the treatment for critically ill COVID-19 patients to reduce the severity of AKI. Regular monitoring of urine output, serum creatinine, avoidance of nephrotoxic drugs, and hemodynamic status is primary management for these patients [11]. Adherence to a lung-protective ventilation strategy is helpful in the reduction of volutrauma and barotrauma-associated kidney injury [12]. Moreover, appropriate maintenance of fluid balance is an essential step to reduce the complications related to volume overloads such as pulmonary edema, ventricular overload, and subsequent AKI [11]. Similarly, hypovolemia should be addressed to prevent the pre-renal type of AKI. However, if conservative treatment is unsuccessful, renal replacement therapy and extracorporeal support should be considered. Continuous renal replacement therapy (CRRT) is also considered for hemodynamically unstable patients [11,13]. Studies have been reported that progression of stages II and III of AKI results in poor prognosis and high mortality rate in patients with renal replacement therapy [13]. Therefore, the need for renal replacement therapy with AKI is an indicator of poor prognosis in a patient with COVID-19 pneumonia [14].

## Conclusions

In summary, we present a unique case report of AKI secondary to COVID-19 pneumonia. Due to its atypical presentation, unlike most other described cases of AKI with COVID-19 pneumonia where patients initially develop COVID-19 pneumonia followed by renal failure makes it an interesting case. This case highlights the importance of early diagnosis, probable underlying pathogenesis, and early initiatives in the mitigation of the complications associated with this disease.
